# Using In-Home Air Quality Monitoring to Reduce Cannabis Secondhand Smoke Exposure in Children: Quantitative Pilot Feasibility Study

**DOI:** 10.2196/89820

**Published:** 2026-06-16

**Authors:** Vincent Berardi, Bradley N Collins, Laura M Glynn, Eusabeia K Silfanus, Darby G Lyons, Stephen J Lepore

**Affiliations:** 1 Department of Psychology, Crean College of Health and Behavioral Science, Chapman University, 1 University Drive, Orange, CA, 92866, United States, 1 (714) 516-5883; 2 Department of Social and Behavioral Sciences, Barnett College of Public Health, Temple University, Philadelphia, PA, United States

**Keywords:** cannabis, secondhand smoke, indoor air monitoring, digital health, self-monitoring

## Abstract

**Background:**

An estimated 5 to 8 million US children live with a parent who uses cannabis, and most cannabis users report smoking cannabis inside their homes, placing children at risk for cannabis secondhand smoke (cSHS) exposure. Indoor air quality (IAQ) monitoring provides real-time feedback on airborne pollutants and has shown promise in reducing in-home tobacco secondhand smoke exposure, suggesting its potential as an effective harm reduction strategy for cSHS.

**Objective:**

This pilot study evaluated the feasibility, acceptability, and preliminary effectiveness of using low-cost, off-the-shelf IAQ monitors to increase caregivers’ awareness of children’s cSHS exposure risk and to change smoking behavior. Secondary aims were to assess participant engagement, perceived usefulness, and household communication regarding in-home cannabis smoking.

**Methods:**

Between February 2025 and April 2025, 14 adults who smoked cannabis indoors and lived with at least 1 child aged younger than 16 years were recruited primarily via targeted social media advertisements and completed a 3-week trial. Participants received an Awair Element IAQ monitor, printed health education materials, and text messaging prompts for brief surveys. The IAQ monitor continuously measured PM_2.5_, VOCs, CO₂, temperature, and humidity. Daily surveys captured self-reported PM_2.5_ readings and recent cannabis use, while baseline and end-of-study assessments evaluated IAQ perceptions, cSHS risk awareness, and in-home smoking behavior. Survey results were summarized via descriptive statistics, and linear mixed-effects models were used to characterize objective IAQ trends. Six additional adult household members provided parallel end-of-study data.

**Results:**

Reported engagement was high, with 85% (11/13) of participants indicating that they reviewed the monitor at least daily. The average number of days in the previous week that a caregiver reported a child being home while cannabis was smoked declined from 4.5 (SD 2.2) at the trial start to 2.8 (SD 2.9) at the end (6/13, 46% had a reduction; 1/13, 8% reported an increase). Furthermore, 62% (8/13) of participants reported that they reduced (4/13, 31%) or thought about changing (4/13, 31%) their smoking habits. Around 62% (8/13) of participants agreed or strongly agreed that IAQ monitoring helped drive conversations about changing indoor smoking rules, while 100% (13/13) reported no IAQ-driven disagreements among household residents regarding in-home smoking rules. A linear mixed-effects model did not indicate a consistent trend in PM_2.5_ levels across participants over time (β=–0.28; SE 1.13; *P*=.81), but there was heterogeneity in trends, and those with the largest reductions in PM_2.5_ over the trial had the largest reduction in reported children’s cSHS exposure.

**Conclusions:**

In-home IAQ monitoring was feasible and perceived as useful among caregivers who smoked cannabis indoors. Real-time IAQ feedback supported risk awareness, promoted family dialogue, and coincided with reductions in in-home smoking around children. These findings suggest that IAQ feedback may represent a scalable tool for reducing children’s cSHS exposure and merits further testing in larger, controlled trials.

## Introduction

An estimated 5.3 to 8 million children in the United States reside with a parent who actively uses cannabis [1], which occurs within the context of increasing cannabis use in the United States [2,3]. Among parents living with children, past-month cannabis use increased by 38.8%, from 4.9% in 2002 to 6.8% in 2015 [1], the most recent year for which national estimates are available. Smoking is the most common method of cannabis use [4], and in-home cannabis smoking is now more prevalent than in-home tobacco smoking [5]. In a nationally representative sample, a majority of cannabis users reported smoking cannabis in their home within the past month (weighted estimate: 54.9%; unweighted: 66.0%) [6], which places other household residents, including children, at risk for cannabis secondhand smoke (cSHS) exposure [7]. Furthermore, validated cSHS biomarkers [8,9] show that among children aged 3 years and younger, the odds of cSHS exposure were 5.02 times higher when their caregiver reported in-home cannabis smoking versus when they did not [10].

While the long-term health impacts of exposure to cSHS are still emerging [11], the well-established risks of tobacco SHS exposure raise concerns that cSHS may pose similar dangers [12]. Cannabis and tobacco smoke have substantial overlap in harmful chemical constituents (eg, volatile organic compounds [VOCs] and carcinogens) [13-15], and both products emit large quantities of airborne PM_2.5_ (fine particulate matter with a diameter <2.5 μm) [16-18]. PM_2.5_ is the pollutant considered most hazardous to global health and the foremost environmental risk factor for cardiovascular disease [19,20]. In addition, cSHS exposure has been linked to increased respiratory symptoms [21], eye irritation [22], and psychoactive effects [23]. Generally, children are more vulnerable to environmental toxins [24-26], which may exacerbate their health risks from cSHS exposure. Accumulating evidence links caregiver cannabis use to child health, including poorer respiratory function [27], increased risk for asthma [28], higher rates of behavior problems [29], and a marginally significant increase in respiratory and allergic or atopic conditions [30]. These findings underscore the need to reduce children’s exposure and highlight the importance of identifying factors that influence caregivers’ efforts to do so.

Increasing parents’ awareness of tobacco secondhand smoke (SHS)-related health risks has been shown to increase their protective behaviors, such as establishing smoke-free home policies [31-38]. A similar dynamic may result from increasing parental awareness of children’s cSHS exposure. For example, adults who rated cSHS as extremely harmful were 6 times more likely to adopt protective behaviors compared to those who considered it completely safe [39]. However, challenges undermine the awareness of cSHS risks. Many smokers lack an accurate understanding of SHS exposure dynamics and report that exposure occurs only when smoke is visible or smelled [40], even though more than 80% of tobacco SHS is invisible to humans [41]. While this misperception has been noted for tobacco, it likely also exists for cannabis and is exacerbated by a prevalent misconception that cSHS is not as dangerous as tobacco SHS [42-44]. Together, these phenomena may reduce caregivers’ urgency to mitigate their children’s cSHS exposure.

Indoor air quality (IAQ) monitoring is a promising strategy to increase parental awareness of cSHS exposure and to prompt efforts to reduce it. Such systems deploy in-home sensors that continuously track airborne pollutants (eg, particulate matter, VOCs, and carbon monoxide), which are likely elevated in homes when cannabis is smoked. Based on tobacco intervention studies that have used IAQ monitoring along with related feedback to reduce SHS exposure, this approach holds great promise for improving cSHS risk awareness among caregivers who smoke cannabis. For example, in a randomized clinical trial conducted with approximately 300 parents who smoked tobacco and lived with at least 1 child, residential IAQ monitoring combined with real-time feedback of PM_2.5_ levels reduced SHS in households by 18.8% [45,46]. A second randomized clinical trial found no evidence that IAQ feedback prompted the creation of smoke-free homes among mothers exposed to SHS, though the observation period was limited to 6 days and participants were not required to be smokers [47].

The purpose of this study, the Cannabis Air Quality Study (C-AIR), was to pilot test the feasibility and acceptability of using off-the-shelf Internet of Things (IoT) air monitors for caregivers who smoke cannabis indoors and live with children. Specific aims included assessing participants’ engagement, perceived usefulness, awareness of cSHS risks, household communication about smoking rules, and smoking behavior change. Where available, data from additional household members were collected to provide broader context on the intervention’s acceptability and utility.

## Methods

### Ethical Considerations

In accordance with federal regulations for the protection of human participants, this study was reviewed and approved by the Chapman University Institutional Review Board (IRB-25‐72). All participants provided informed consent before enrollment, and all data were deidentified for analysis to maintain participant confidentiality. Participants who fully completed the study were paid US $120 in compensation, which was provided at 3 study time points.

### Participants and Recruitment

*Primary participants* were recruited via targeted digital advertisements on Facebook and Instagram between February 12, 2025, and April 28, 2025. As shown in Multimedia Appendix 1, ads were created using Meta’s Ad Manager tool, which tailors content to optimize engagement across various components of their ecosystem (Facebook Ads, Instagram Reels, phone vs computer, and so on). We also identified cannabis-related forums on Reddit and contacted the moderators to provide details about the study and inquire whether their platform would be an appropriate venue for recruitment. If a positive response was received, we created a post with a PDF version of the Meta advertisement and a clickable link. Finally, the researchers recruited 2 participants from their extended personal network. Fourteen adults were enrolled according to the following inclusion criteria: (1) aged at least 21 years; (2) resided in California; (3) spoke English; (4) had a smartphone and in-home WiFi; (5) reported smoking cannabis indoors at least once per week, with bioverification of cannabis use; and (6) lived with at least one child aged younger than 16 years.

To capture the experiences of household members not directly involved in the intervention, each primary participant was invited, at the end of the trial, to identify 1 adult household member to serve as a *household participant* respondent. Six respondents were enrolled (a maximum of one per household), and each completed a survey regarding their experiences.

### Study Overview

Participants completed screening, provided informed consent, underwent bioverification, and participated in a baseline assessment prior to receiving an IAQ monitor for a 3-week intervention period. During the study, participants completed brief daily or near-daily surveys that assessed their ability to interpret monitor readings and their recent smoking behaviors. At the conclusion of the intervention, participants and, when available, 1 additional adult household member completed end-of-study assessments regarding experiences, behaviors, and perceptions of cSHS.

### Study Procedures

Eligible respondents were identified via an initial screening survey and completed a 20-minute enrollment call, during which study procedures were reviewed, informed consent was obtained, and a baseline assessment video call was scheduled. Participants were then mailed an in-home cannabis urine bioverification kit (Easy@Home Marijuana Urine Test, Easy Healthcare Corp) and were instructed to complete the test 5 minutes prior to their scheduled baseline assessment. At the start of the call, participants displayed the test result on camera for interviewer verification. Participants with confirmed cannabis use completed a baseline questionnaire administered via a computer-assisted telephone interview conducted through Qualtrics, which assessed demographics, perceptions of IAQ, awareness of cSHS risks, smoking behaviors, and attitudes toward secondhand smoke mitigation. All participants received a US $30 Amazon gift card for completing the bioverification, regardless of the result.

Following the baseline assessment, participants were mailed an IAQ monitor kit that included (1) an Awair Element [48] air monitor (Bitfinder, Inc), (2) printed installation and usage instructions, (3) an information sheet describing the health risks of PM_2.5_ and its presence in cSHS (Multimedia Appendix 1), and (4) return packaging with prepaid postage. The Awair Element is an off-the-shelf IoT IAQ monitoring device that continuously measures PM_2.5_, total VOCs, CO₂, temperature, and humidity. It features onboard WiFi for automatic cloud-based data upload, a large alphanumeric display that shows real-time IAQ readings, and a companion mobile app that allows participants to access real-time and historical data. Awair also provides a cloud-linked dashboard that serves as a centralized system where C-AIR study personnel could monitor device connection status and current or historical IAQ data from all deployed devices.

Participants were instructed to install the device in the primary living area where cannabis smoking typically occurred. Device connectivity was monitored through the Awair dashboard, and if installation was not confirmed within 24 hours of delivery (as tracked via the United States Postal Service), a reminder text was sent. For the small number of participants (n=4) who required installation support, sending a recorded video guide resolved the issue in every case. Upon confirmation of successful installation, participants received an additional US $30 Amazon gift card. To encourage regular engagement with the IAQ monitor and promote an accurate understanding of its functionality and output, participants received automated SMS text messaging prompts via REDCap (Research Electronic Data Capture; Vanderbilt University) at 7 PM daily during the first week of the study and every other day during the remainder of enrollment. Each message included a link to a brief survey asking participants to report (1) the highest PM_2.5_ value observed that day, as highlighted within the Awair companion app; (2) the time of their most recent indoor cannabis use; and (3) the time of their most recent indoor tobacco use (for dual cannabis and tobacco users only).

At the conclusion of the 3-week intervention, participants completed a computer-assisted telephone interview-administered end-of-study questionnaire assessing their experiences with the air monitor, recent smoking behaviors, perceptions of cSHS, and other relevant feedback. A US $60 Amazon gift card was provided upon completion of this assessment, bringing the total compensation for the study to US $120. Participants were instructed to return the Awair monitors using the prepaid shipping materials provided. SMS text messaging reminders were sent if the device was not received within 7 days of the final assessment.

Following the end-of-treatment assessment, participants were asked whether any other adult household member would be willing to enroll as a household participant and complete a survey about their experiences. Interested individuals were sent a link to a screening survey and were instructed to forward it to the other household member. Household membership was confirmed by cross-referencing the self-reported age, gender, and first initial of each respondent with household composition information provided by the primary participant at the outset of the trial. Once eligibility was confirmed (see “Participants and Recruitment” section for inclusion criteria), a link was sent to a self-administered online survey that assessed behaviors and patterns regarding substance use, attitudes toward the air monitor, and family dynamics around IAQ monitoring. Household participants received a US $30 Amazon gift card upon survey completion.

### Study Measures

Assessment measures were drawn from multiple sources. Cannabis use frequency and patterns were assessed using items adapted from the Daily and Future Assessment of Cannabis Use [49]. Motivation to quit was assessed via the single-item Motivation to Stop Scale [50]. The severity of cannabis and nicotine addiction was based on the Fagerström Test [51]. Items assessing IAQ perceptions, household smoking behaviors, and end-of-study process variables were developed specifically for this study by a research team with extensive expertise in secondhand smoke exposure and behavioral intervention research, with select measures drawn from their previous studies [52,53]. All measures were administered at baseline, with a subset of items repeated at the end of the study to assess change over time. Responses were captured using a combination of Likert-type scales, ordinal response options, and numeric sliders. The full survey instrument is shown in Multimedia Appendix 2.

### Statistical Analyses

Given the small sample size for this pilot study, our analytic approach primarily consisted of descriptive statistics of responses to selected questions. Cross-tabulations were used to assess the relationship between perceptions of in-home smokiness and children’s exposure to cSHS at both the beginning and end of the study. Differences at the beginning and end of the trial in self-reports of children’s presence in the home while cannabis was smoked were recorded as a numeric change, and they were assessed via a paired 2-sample 2-tailed *t* test. These changes were cross-referenced with perceptions of in-home smokiness and children’s exposure to cSHS (descriptive statistics only, due to the small sample size).

To examine individual trajectories and overall patterns in objectively measured IAQ, PM_2.5_ values were aggregated at the day level for each participant. Self-reported PM_2.5_ measures were only used to assess engagement. Preliminary analyses using nonparametric smoothing indicated that trajectories were approximately linear, so we opted for a linear mixed-effects model with fixed effects for time (ie, day) and random intercepts and slopes for each participant. This approach captures both average trends and individual variation in the rate of change. Three participants were excluded from this analysis due to a data logging error that generated missing IAQ data during their first week of enrollment. Data from the remaining 11 participants were modeled over their first 18 days of enrollment in order to retain the largest possible analytic sample, as 2 participants’ observation periods ended after days 18 and 19 due to attrition. This approach was then repeated for the Awair score, which is a proprietary metric of IAQ that considers all features (contamination, temperature, humidity, and so on) and is highlighted by default in the Awair app. Changes in PM_2.5_ values were also cross-referenced with self-reports of children being present in the home while cannabis was smoked. The mixed-effects models were estimated using the *lme4* package in R software (R Foundation for Statistical Computing) with restricted maximum likelihood.

## Results

### Recruitment and Retention

As shown in Table 1, C-AIR social media advertisements generated 104,500 views and 2891 clicks (US $0.29/click), resulting in 278 completed screening surveys. Of these, 38 respondents met eligibility screening criteria; 15 advanced to the consent or bioverification stage, while the remaining eligible respondents were lost prior to consent due to nonresponse or failure to schedule a consent appointment. Bioverification of cannabis use was positive for all participants, but 1 participant was dropped after failing to confirm that they lived with a child aged younger than 16 years during the baseline assessment. In total, 14 participants were enrolled, with one unable to complete the end-of-trial assessment due to a family emergency. All but one air monitor was successfully returned.

**Table 1.** Participant flow from digital impressions for a targeted social media campaign through the analytic sample^a^^b^.

Views	Clicks	Completed screening survey	Eligible	Enrolled	Completed all study surveys	Sufficient data for IAQ^c^ modeling
104,500	2891	278	38	14	13	11

a One participant was unable to complete the end-of-study assessment due to personal circumstances, but remained actively engaged throughout the monitoring period.

b Sufficient data for IAQ modeling was defined as having recorded PM_2.5_ data over the first 18 days of enrollment. PM_2.5_ is the fine particulate matter with a diameter <2.5 µm.

c IAQ: indoor air quality.

### Participant Demographics

Table 2 shows the demographics of study participants. They were primarily female, non-White, and college graduates, with an average age of 42.3 (SD 11.2) years and an average household income of US $67,714 (SD US $42,707). The mean age of the youngest child in the home was 11.3 (4.5) years. Nearly 80% of participants reported smoking cannabis multiple times per day over the past month, and on days when cannabis was smoked, an average of 3.4 (SD 2.6) cannabis smoking events occurred in the home.

**Table 2.** Participant characteristics (N=14).

Characteristics	Values
Age, mean (SD)	42.3 (11.2)
Youngest child’s age, mean (SD)	11.3 (4.5)
Gender, n (%)	
Female	10 (71)
Male	4 (29)
Race or ethnicity, n (%)^a^	
Asian	1 (7)
Black	2 (14)
Middle Eastern	1 (7)
Mixed race or ethnicity	3 (21)
White	6 (43)
No response	1 (7)
College degree or higher, n (%)	12 (86)
Currently employed, n (%)	9 (64)
Household income (US $), mean (SD)	67,714 (42,707)
Another adult lives in home, n (%)	11 (79)
Own residence, n (%)	5 (38)
Tobacco smoker, n (%)	6 (43)
Smoke cannabis multiple times per day, n (%)	11 (79)
Number of In-home cannabis events on typical day you smoke, mean (SD)	3.4 (2.6)
Desire to quit cannabis, n (%)	
“I don’t want to quit”	10 (71)
“I think I should, but don’t want to quit”	2 (14)
“I want to quit and hope to soon”	1 (7)
“I want to stop but haven’t thought about when”	1 (7)
Primary method of cannabis administration, n (%)	
Joints or blunts	9 (64)
Pipe or water pipe or bong	3 (21)
Vaporizer	2 (14)

a Race or ethnicity was collected via open-ended self-report and subsequently coded into categories.

### Engagement with and Accuracy in Interpreting IAQ Data

Participants regularly reviewed IAQ data, with 85% (11/13) indicating that they reviewed the monitor at least daily, including 38% (5/13) who reviewed it multiple times per day. Furthermore, 77% (10/13) of participants reported a consistent level of engagement throughout the trial.

A total of 192 daily surveys were distributed to primary participants (14 per participant, minus 4 that were not sent due to a software error), with 166 (86%) completed. The survey asked participants to report the highest PM_2.5_ concentration over the past 24 hours, as highlighted within the Awair companion app. Reported values were compared with those logged by the IAQ monitor so that accuracy could be assessed. Across all responses, 36.5% were accurate (exact matches), with a mean/median participant-level match rate of 36.9%/40.3%. Five participants had no exact matches, while 4 achieved matches on 75% or more of their responses, indicating substantial heterogeneity in the accuracy of participants’ app reviews.

### Perceived Usefulness and Impact on cSHS-Related Household Communication

At the conclusion of the trial, participants indicated that the IAQ monitor was useful, with a large proportion strongly agreeing that they would recommend the device to cannabis smokers trying to reduce cSHS in their homes (10/13, 77%) or reduce their cannabis smoking (9/13, 69%).

Many participants also reported that the IAQ monitor facilitated constructive conversations around indoor smoking. Specifically, 54% (7/13) strongly agreed and 15% (2/13) agreed that the IAQ monitoring helped drive conversations about changing indoor smoking rules. Importantly, of the 10 participants who live with at least 1 other adult, 90% (9/10) strongly disagreed and 10% (1/10) disagreed with the statement that the IAQ monitor led to disagreements among household residents regarding in-home smoking rules.

### Changes in Self-Reported In-Home Cannabis Smoking Behavior

Beyond facilitating discussions, we find evidence that IAQ monitoring may also influence caregivers’ indoor cannabis smoking practices. Many participants reported modifying their in-home smoking behavior during the monitoring period. In particular, the average number of days in the previous week that a caregiver reported a child was in the home while cannabis was smoked declined from 4.5 (SD 2.2) at the beginning of the trial to 2.8 (SD 2.9) at the end (*P*=.14; Cohen *d*=0.46; n=13). At baseline, no participants reported 0 days in the past week during which a child was home while cannabis was smoked. By the end of the trial, 4 (28.6%) reported 0 days, including 2 of the 5 participants who had reported every day at baseline.

More broadly, IAQ monitoring coincided with shifts in general cannabis smoking behavior, independent of whether smoking occurred indoors or around children. Around 62% (8/13) of participants reported that they either reduced (4/13, 31%) or thought about changing (4/13, 31%) their cannabis smoking habits during the study. Furthermore, when asked to estimate the percentage of all cannabis smoking events over the past week that occurred inside the home, there was a modest decrease from 77.2% to 70.0%. No discernible pattern was observed between the desire to quit cannabis and any of these outcomes.

### Changes in Perceived IAQ and cSHS Exposure Risk

IAQ monitoring was also associated with changes in participants’ perceptions of their home’s IAQ and children’s exposure risk. For instance, Table 3 depicts the self-reported smokiness of the home at the beginning and end of the study. Over half (7/13) of the participants changed their views of their home’s smokiness over the course of the trial, with 57% (4/7) reporting improvements in IAQ by the study’s end. Those reporting improved IAQ had the most in-home cannabis events with a child present at baseline and the largest reduction at the end of the trial. Those whose perceptions of IAQ did not change had more modest reductions in in-home cannabis events with a child present, while those who felt their IAQ had worsened experienced no change.

**Table 3.** Perceived home smokiness and associated in-home cannabis smoking behavior among caregivers who smoke cannabis indoors and have completed all surveys (N=13).

Response	Baseline	End of treatment
How smoky is your home? n (%)		
Not at all	2 (15)	3 (23)
A little	5 (38)	7 (54)
Somewhat	4 (31)	1 (8)
Very	1 (8)	2 (15)
Do not know	1 (8)	0 (0)
Number of in-home smoking events over past week with a child at home, stratified by the direction of change in perceived smokiness from baseline to end-of-treatment (n=12)		
Increased perceived smokiness (n=2)	4.0 (1.4)	4.0 (1.4)
Consistent perceived smokiness (n=6)	3.2 (2.0)	1.8 (2.7)
Decreased perceived smokiness (n=4)	7.0 (0.0)	3.5 (4.0)

a One participant could not be classified as increased, consistent, or decreased perceived smokiness since they reported that they did not know how smoky their home was at baseline.

In addition to general air quality perceptions, we compared parents’ baseline versus end-of-study reports of children’s risks of cSHS exposure (Table 4). Both participants who initially indicated that their children were at no risk reported elevated risk at the trial’s conclusion. Among the 4 participants who initially rated their children as very much at risk, 3 perceived lower risk at follow-up, with 2 reporting no risk at all. Of those who reported their children were somewhat at risk, 57% (7/ 4/7) continued to believe this at the end of the trial, while 43% (3/7) perceived less risk. Importantly, reductions in perceived cSHS risk were accompanied by markedly lower numbers of in-home cannabis smoking events with a child present. In contrast, those whose risk perceptions increased or remained unchanged showed essentially no change in smoking events.

Given the small subgroup sizes in this section, these findings should be interpreted with caution.

**Table 4.** Perceived children’s risk of cannabis secondhand smoke (cSHS) exposure and associated in-home cannabis smoking behavior among caregivers who smoke cannabis indoors and completed all surveys (N=13).

Response	Baseline	End of treatment
How at risk are your children to cSHS exposure? n (%)		
No risk at all	2 (15)	5 (38)
Somewhat at risk	7 (54)	7 (54)
Very much at risk	4 (31)	1 (8)
Number of in-home smoking events over past week with a child at home, stratified by the direction of change in perceived child risk from baseline to end-of-treatment, mean (SD)		
Increased perceived risk (n=2)	6.0 (1.4)	6.0 (1.4)
Consistent perceived risk (n=5)	4.0 (2.7)	4.2 (2.6)
Decreased perceived risk (n=6)	4.6 (2.3)	0.5 (1.2)

### Objective IAQ Measures

**Figure 1.**
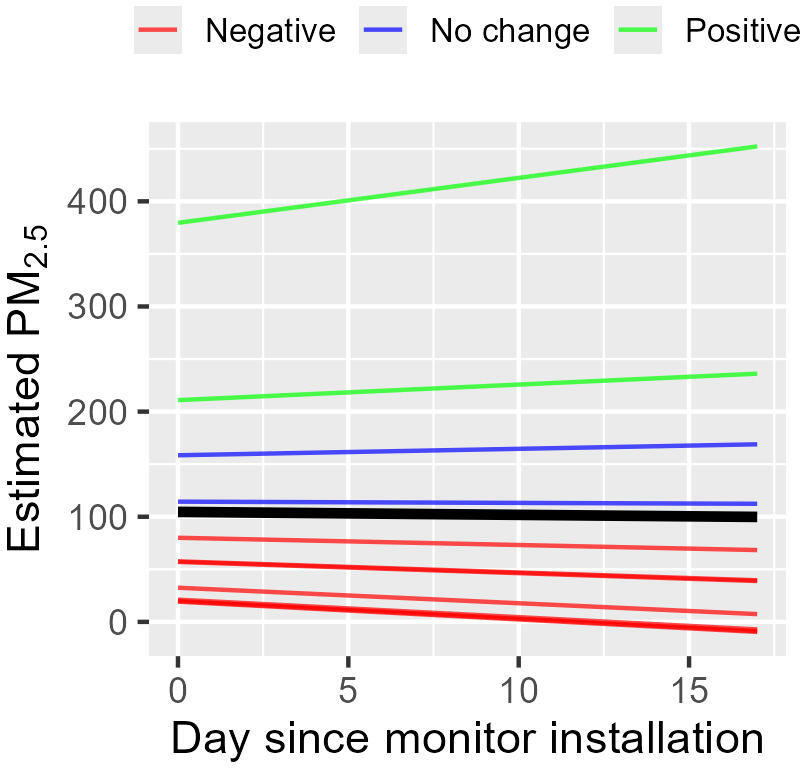
Estimated participant-specific trajectories over the intervention period from linear mixed-effect models of PM_2.5_ daily means for each participant, with the thick black line representing the fixed effect. The remaining trajectory colors reflect the direction and magnitude of change in the estimated values from day 0 to 17: green indicates an increase of greater than 10%, red indicates a decrease of greater than 10%, and blue indicates a change of less than 10%. PM_2.5_: fine particulate matter with a diameter of less than 2.5 µm.

The linear mixed-effects model indicated no consistent trend in levels across participants over time for either PM_2.5_ (β=–0.28; *P*=.81) or Awair score (*β*=0.02; *P*=.91). However, there was substantial individual variability in baseline levels and rates of change, as reflected in the random intercept and slope terms. This pattern is consistent with the study design, in which air monitors began recording immediately upon installation without a baseline or run-in period, likely contributing to individualized reactivity. Figures1  2 illustrate the participant-specific trajectories, highlighting the heterogeneity in PM_2.5_ and Awair score patterns across individuals.

Individual participant trajectories were also cross-referenced with participants’ perceptions and self-reported behaviors. The 6 participants with a greater than 10% decrease in PM_2.5_ measures had the largest reduction in the average number of self-reported in-home cannabis events with children present (−3.33 vs 0 for no change in PM_2.5_ and −2 for increasing trajectories), thereby validating their observations of reduced indoor cannabis smoking episodes. Furthermore, among participants whose PM_2.5_ trajectories increased or remained consistent, 50% (2/4) never accurately reported their highest PM_2.5_ reading on a daily survey, compared to 6 of 7 participants with decreasing trajectories who had at least 1 accurate reading, suggesting that the ability to accurately read the monitor may be a key mechanism linking IAQ feedback to behavior change. Among participants with decreasing PM_2.5_ trajectories, 4 of 6 also reported reduced perceptions of children’s cSHS exposure risk, though the small subgroup sizes preclude firm conclusions.

**Figure 2.**
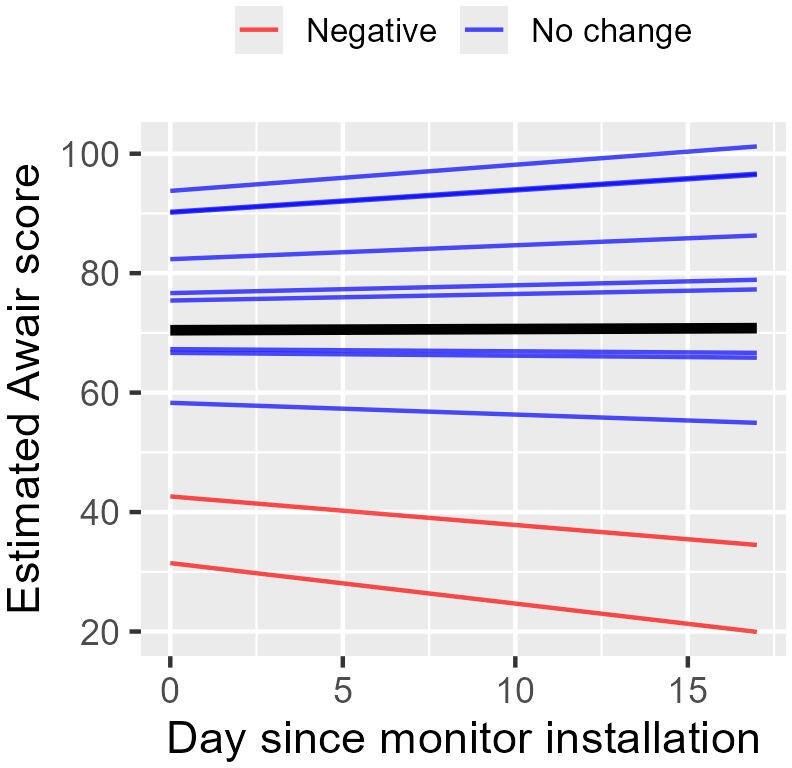
Estimated participant-specific trajectories over the intervention period from linear mixed-effect models of Awair score daily means for each participant, with the thick black line representing the fixed effect. The remaining trajectory colors reflect the direction and magnitude of change from day 0 to day 17: red indicates a decrease of greater than 10%, and blue indicates a change of less than 10%.

### Household Participant Reports

Six households had an additional adult who completed a questionnaire as a household participant. Four of these individuals were a spouse or significant other of the primary participant, while one was an adult child and the other was a roommate. Five smoked cannabis every day or most days, while one smoked on some days. As was the case with the primary participants, household participants updated their perceptions of their home’s IAQ with a trend toward poorer IAQ at the outset of the study than they initially suspected. More specifically, 4 out of 6 (67%) participants retrospectively reported poorer IAQ at the beginning of the survey compared to their current IAQ. Around 4 of 6 (67%) of household participants either agreed or strongly agreed with the statement that the IAQ monitoring was helpful for family conversations about smoking rules. About 3 of 6 (50%) either disagreed or strongly disagreed with the statement that IAQ monitoring led to disagreements about household smoking rules, with 2 of the remaining 3 participants indicating that this question was not applicable, likely due to no discussions occurring.

## Discussion

### Summary of Findings

This pilot study demonstrates that in-home IAQ monitoring was feasible among caregivers who smoke cannabis indoors. Participants reported reductions in in-home cannabis smoking while children were present, increased perceptions of children’s cSHS risks, increased household communication about smoking rules, and high perceived usefulness of the devices. Together, these findings provide preliminary evidence that a brief IAQ monitoring intervention can prompt meaningful behavioral and attitudinal change in this population without introducing interpersonal conflict to the household.

### Interpretation and Comparison With Prior Work

The largest decreases in indoor cannabis smoking were observed among those who, after IAQ monitor deployment, also reported the greatest perceived improvements in air quality and reductions in children’s cSHS risk. These trends suggest that IAQ feedback may facilitate harm-awareness and harm-reduction behaviors within the household. However, among the slightly more than half of participants who reported no change or worsening air quality and cSHS risk, there was little evidence of change in the number of in-home cannabis smoking events while children were present. This pattern may indicate limitations in the effectiveness of pairing IAQ monitoring with only brief written health education provided at the study’s initiation. Based on evidence from tobacco SHS reduction trials, it is likely that more intensive counseling paired with IAQ monitoring would be more effective in generating reductions in cSHS. Future work should explore how additional behavioral or educational supports might amplify the impact of IAQ feedback.

Most participants found the IAQ monitors useful and—critically—reported that the devices helped drive conversations about changing indoor smoking rules. Notably, the intervention did not generate household conflict around cSHS exposure and efforts to mitigate children’s risk, an important consideration when designing family-centered public health interventions. This observation suggests that IAQ monitoring can facilitate constructive, data-driven discussions about exposure, which could be particularly valuable in homes where power dynamics might otherwise stifle such conversations.

Low-cost, IoT-enabled IAQ monitoring has emerged as a feasible and accessible approach for residential settings, with recent systematic reviews confirming the viability of consumer-grade sensors for home-based air quality assessment [54]. Early work demonstrated that visualizing IAQ data within social networks increased awareness, prompted behavior change, and fostered collaborative household efforts to improve air quality [55]. Subsequent feasibility work confirmed that IAQ monitoring was acceptable and useful among parents in households with SHS exposure [56]. This approach has also shown promise in health-focused apps, with studies demonstrating the feasibility of pairing low-cost IAQ monitors with ecological momentary assessment in homes with children who have asthma [57] and documenting high usability and positive participant feedback when using devices to track residential PM_2.5_ in underserved populations [58]. In tobacco-focused SHS interventions specifically, IAQ monitoring paired with personalized feedback has been found to be both feasible and acceptable among caregivers who smoke indoors with young children, with participants identifying personalized exposure feedback as a key motivator for behavior change [59]. This study extends this body of work to the underexplored context of residential cannabis smoking and children’s cSHS, where such tools remain largely untested. Furthermore, the current off-the-shelf IAQ technology used in this project, similar to that deployed by Cavalier et al [58], represents a substantial advancement in ease of use and overall user experience compared to the real-time IAQ systems deployed in previous tobacco-focused studies and interventions. These improvements, together with the project’s demonstrated success in remotely enrolling participants and bioverifying cannabis use, suggest that this approach could be scalable for future efficacy and implementation trials.

### Limitations

This study has several limitations. The small sample size and lack of a control group limit generalizability and prevent strong causal claims. This is particularly true for subgroup findings, which should be considered exploratory and are most appropriate for hypothesis generation. As is common with targeted social media recruitment, uptake among those who encountered study advertisements was low, which has implications for the scalability of this recruitment approach. A data logging error resulted in the exclusion of 3 participants from the IAQ modeling analysis, further reducing an already small analytic sample. Outside of the air monitors, all outcome data were self-reported and may be subject to recall or social desirability bias. However, the consistency of patterns across multiple domains lends credibility to the findings. In addition, our study captured a relatively short time window, and longer-term impacts on behavior and sustained device engagement remain unknown. Finally, because there was no baseline IAQ measurement period during which participants were blinded to monitor readings, reactivity to the device precluded the IAQ measures from serving as an objective outcome measure.

### Conclusions

Our findings add to a growing body of evidence suggesting that cSHS may warrant comparable public health attention. The lack of regulatory messaging and widespread misperceptions around cSHS risks creates a clear need for tools that can bridge this gap. IAQ monitoring is well-positioned to serve as one such tool, particularly because it allows for nonjudgmental, data-driven engagement with smokers who may not otherwise view their behavior as harmful. This pilot study demonstrates that low-cost, off-the-shelf IAQ monitors are feasible to install in cannabis-using households and potentially impactful for reducing children’s exposure to cSHS. Participants engaged with the devices, perceived them as useful, and reported meaningful shifts in behavior and family dialogue. These findings suggest that even simple environmental feedback tools can serve as powerful behavioral cues, offering a scalable strategy for addressing cSHS in homes where children are present.

Future studies should build on this work by enrolling larger samples to improve generalizability and explore whether IAQ monitoring is equally effective across a range of household contexts. There is also a need to develop counseling protocols that incorporate real-time IAQ feedback to help families identify specific times, triggers, or contexts associated with cSHS exposure, which enables the creation of personalized harm-reduction strategies. Embedding IAQ monitoring into existing systems, such as pediatric care, primary care visits, and/or cessation counseling, could amplify its reach and normalize its use in both clinical and community settings. Importantly, these efforts should be pursued in close collaboration with community stakeholders to ensure that cannabis users can help shape how these technologies are introduced, interpreted, and acted upon in their own lives. Such co-design processes will increase the relevance, acceptability, and sustainability of IAQ-based interventions [60,61].

## Supplementary material

10.2196/89820 Multimedia Appendix 1 Recruitment and study materials.

10.2196/89820 Multimedia Appendix 2 Assessment survey.

## Abbreviations

cSHS: cannabis secondhand smoke
IAQ: indoor air quality
IoT: internet-of-things
PM_2.5_: fine particulate matter
SHS: secondhand smoke
VOC: volatile organic compound
