# Supplementary material for: Using In-Home Air Quality Monitoring to Reduce Cannabis Secondhand Smoke Exposure in Children: Quantitative Pilot Feasibility Study
Source: JMIR Form Res. 2026 Jun 16;10:e89820. doi: 10.2196/89820 (PMC13271585; doi:10.2196/89820)

Social Media Recruitment Ad

(*Note: Ad content slightly varied across platforms via tweaks automatically made by the Meta system)*


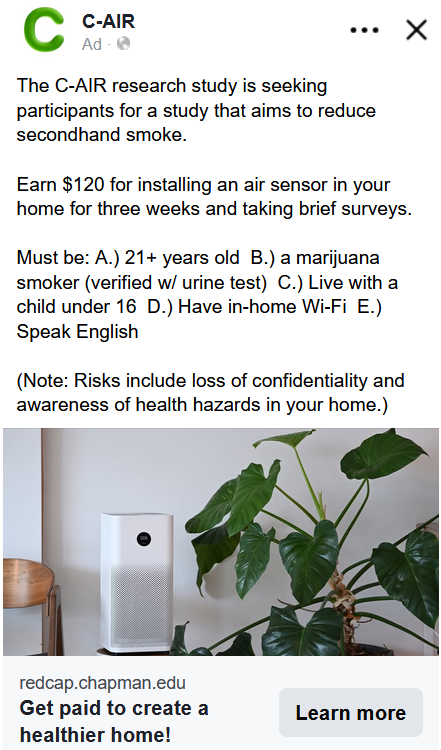


Educational Information Sheet Provided to Participants


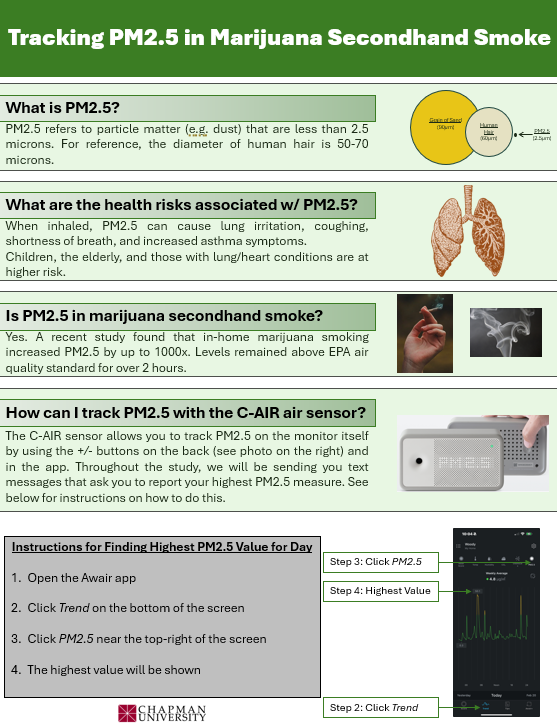

Supplement: Multimedia Appendix 1 [file formative-v10-e89820-s001.docx]
