# Supplementary material for: Using In-Home Air Quality Monitoring to Reduce Cannabis Secondhand Smoke Exposure in Children: Quantitative Pilot Feasibility Study
Source: JMIR Form Res. 2026 Jun 16;10:e89820. doi: 10.2196/89820 (PMC13271585; doi:10.2196/89820)
Supplement: Multimedia Appendix 2 [file formative-v10-e89820-s002.docx]

| *Item* | *Response Options* | *Condition For Display* |
| --- | --- | --- |
| **Demographics** | | |
| Participant uses only marijuana or marijuana & tobacco? (from screening form) | Marijuana only; marijuana + tobacco | Always |
|  |  |  |
| What is your age? | Open text | Baseline only |
| What is your gender? | Male; Female; Non-binary / third gender; Prefer not to say; Other: ____ | Baseline only |
| Do you consider yourself Hispanic or Latino? | Yes; No | Baseline only |
| How would you describe your race? | Open text | Baseline only |
| What is your current marital status? | Married or Domestic Partnership; Single (including widowed, separated, divorced) | Baseline only |
| What is the highest level of education you have completed | Less than high school; High school graduate, or equivalent; College graduate (2 or 4 year degree); Graduate degree | Baseline only |
| What is your current employment status? | Employed (includes full-time, part-time, student); Unemployed (includes looking and not looking for work, retired, homemaker) | Baseline only |
| What is your annual household income? | Open text | Baseline only |
| Do you rent or own your residence | Rent; Own | Baseline only |
| Which of the following best match the smoking rules put in place by your landlord? | No smoking allowed inside the home and this policy is enforced; No smoking allowed inside the home and this policy is not enforced; Smoking is allowed inside the home | If participant rents residence |
| Which of these match the best smoking rules in your home? | No smoking allowed inside the home and this policy is enforced; No smoking allowed inside the home and this policy is not enforced; Smoking is allowed inside the home | If participant owns residence |
| How many adults 18 and over, including yourself, live in the home? | Numeric slider 1-10 | Baseline only |
| For each adult in the home, can you please tell me their: Age; Gender; If they smoke marijuana, cigarettes, both, or none; First intial | Open text | If number of adults > 1 |
| How many children <16 years of age live inside the home? | Numeric slider 1-10 | Baseline only |
| What is the age of the youngest child? | Numeric slider 1-16 | If children present |
| What is the gender of the youngest child? | Male; Female; Non-binary | If children present |
| **Cannabis Use** | | |
| How old were you when you first tried marijuana? | Numeric slider 8-60 | Always |
| Do you use it for medicinal or recreational purpose or both? (select the boxes that apply) | Medicinal; Recreational | Always |
| Do you have a physician's recommendation to use marijuana right now? | No; Yes | If Medicinal selected |
| Looking at the past month, which of the following best describes how often you've smoked marijuana? | 2 – 3 times a month; Once a week; A few times a week; Once a day; Multiple times a day | Always |
| Which of the following best describes you, in terms of marijuana smoking? | I don’t want to stop smoking marijuana; I think I should stop smoking marijuana but don’t really want to; I want to stop smoking marijuana but haven’t thought about when; I really want to stop smoking marijuana but I don’t know when I will; I want to stop smoking marijuana and hope to soon; I really want to stop smoking marijuana and intend to in the next 2 to 3 months; I really want to stop smoking marijuana and intend to in the next month | Always |
| On a typical day when you smoke marijuana, how many times do you smoke inside the home? | Numeric slider 0-10 | Always |
| Out of all the times you've smoke marijuana in the past month, what percentage would you estimate are smoked inside of your home? | Numeric slider 0-100 | Always |
| Out of all the times you've smoked marijuana in your home in the past month, what percentage of times would you estimate that you smoked with someone else? | Numeric slider 0-100 | Always |
| How soon after you wake up do you typically smoke marijuana? | Within 5 minutes; 6 minutes to an hour; 1-3 hours; More than 3 hours | Always |
| Which of the following ways do you smoke marijuana? Select all that apply | Joints; Blunts; Pipe; Bong/Water Pipe; Hookah; Vaporizer; Other: ____ | Always |
| Which one of these is the primary or main way that you smoke marijuana? Select only one | Joints; Blunts; Pipe; Bong/Water Pipe; Hookah; Vaporizer; Other: ____ | Always |
| In a typical month, how often do you take edibles? | Never; 1-3 times per month; Once per week; A few times a week; Once a day; Multiple times a day | Always |
| Would you be open to replacing some of your marijuana smoking with edibles? | Yes; No | Always |
| During the past year did you ever wish you could stop your use of marijuana? | Never/almost never; Sometimes; Often; Always/nearly always | Baseline only |
| **Tobacco Use** | | |
| How soon after you wake up do you first smoke tobacco? | Within 5 minutes; 6 minutes to an hour; 1 to 3 hours; After 3 hours | If marijuana and tobacco use |
| Do you find it difficult to refrain from smoking tobacco where it is forbidden, e.g. church, library, movie theater? | No; Yes | If marijuana and tobacco use |
| Looking at the past month, which of the following best describes how often you smoke cigarettes? | 2-3 times a month; Once a week; A few times a week; Once a day; Multiple times a day | If marijuana and tobacco use |
| On a typical day, how many cigarettes a day do you smoke? | 5 or less; 6-10; 11-20; 21-30; >30 | If marijuana and tobacco use |
| On a typical day, how many times do you smoke tobacco inside the home? | Numeric slider 0-10 | If marijuana and tobacco use |
| Out of all the times you’ve smoked tobacco in the last month, estimate the percentage that were smoked inside of your home? | Numeric slider 0-100 | If marijuana and tobacco use |
| Out of all the times you’ve smoked tobacco in your home in the past month, what percentage of times would you estimate that you smoked with someone else? | Numeric slider 0-100 | If marijuana and tobacco use |
| Do you smoke tobacco more frequently during the first hours after waking than the rest of the day? | No; Yes | If marijuana and tobacco use |
| Do you smoke tobacco if you are so sick that you are in bed most of the day? | No; Yes | If marijuana and tobacco use |
| Which of the following best describes you, in terms of tobacco smoking? | I don’t want to stop smoking tobacco; I think I should stop smoking tobacco but don’t really want to; I want to stop smoking tobacco but haven’t thought about when; I really want to stop smoking tobacco but I don’t know when I will; I want to stop smoking tobacco and hope to soon; I really want to stop smoking tobacco and intend to in the next 2 to 3 months; I really want to stop smoking tobacco and intend to in the next month | If marijuana and tobacco use |
| **Home Smoking Characteristics** | | |
| How would you best describe the rules for smoking marijuana in your house versus the rules for smoking tobacco in your house? | I have stricter rules for smoking tobacco inside my home than I do for smoking marijuana; I have stricter rules for smoking marijuana inside my house than I do for tobacco; My rules for smoking tobacco and marijuana inside my home are the same | Always |
| Considering all sources of smoke in your home over the past 3 days (marijuana, tobacco, fireplace, stove), how smoky was your home? | Not at all; A little; Somewhat; Very; Unknown | Always |
| Thinking back to the beginning of the study, how smoky do you think your home was then? | Not at all; A little; Somewhat; Very; Unknown | EOT only |
| How many days in the past week was your child in the house when someone smoked marijuana? | Numeric slider 0-7 | Always |
| How many days in the past week was your child in the house when someone smoked tobacco? | Numeric slider 0-7 | If marijuana and tobacco use |
| Secondhand smoke is the smoke from burning marijuana or tobacco that can be inhaled by the smokers and others. How much do you believe that your children are at risk from secondhand marijuana smoke in your home? | No risk at all; Somewhat at risk; Very much at risk | Always |
| How much do you believe that your children are at risk from secondhand tobacco smoke in your home? | No risk at all; Somewhat at risk; Very much at risk | If marijuana and tobacco use |
| How much do you want to eliminate your child’s exposure to secondhand marijuana smoke in your home? | Not at all; A little; Very much | Always |
| How much do you want to eliminate your child’s exposure to secondhand tobacco smoke in your home? | Not at all; A little; Very much | If marijuana and tobacco use |
| How confident are you that you can eliminate marijuana secondhand smoke in your home? | Not at all; A little; Very much | Always |
| How confident are you that you can eliminate tobacco secondhand smoke in your home? | Not at all; A little; Very much | If marijuana and tobacco use |
| Thinking back to the beginning of the study, how at risk do you think children in your home were to being exposed to secondhand marijuana smoke in your home then? | No risk at all; Somewhat at risk; Very much at risk | EOT only |
| Thinking back to the beginning of the study, how at risk do you think children in your home were to being exposed to secondhand tobacco smoke in your home then? | No risk at all; Somewhat at risk; Very much at risk | EOT only; If marijuana and tobacco use |
| In terms of health risks for yourself, how does smoking one marijuana joint per day compare with smoking one cigarette per day? | Smoking one marijuana joint a day is much less safe than smoking one cigarette a day; Smoking one marijuana joint a day is somewhat less safe than smoking one cigarette a day; Smoking one marijuana joint a day is as safe as smoking one cigarette a day; Smoking one marijuana joint a day is somewhat safer than smoking one cigarette a day; Smoking one marijuana joint a day is much safer than smoking one cigarette a day | Always |
| How does secondhand smoke from marijuana compare to secondhand smoke from tobacco? | Secondhand smoke from marijuana is much less safe than secondhand smoke from tobacco; Secondhand smoke from marijuana is somewhat less safe than secondhand smoke from tobacco; Secondhand smoke from marijuana is as safe as secondhand smoke from tobacco; Secondhand smoke from marijuana is somewhat safer than secondhand smoke from tobacco; Secondhand smoke from marijuana is much safer than secondhand smoke from tobacco | Always |
| **Process Measures** | | |
| After the air quality monitor was installed, I told other family members and visitors about it | Strongly agree; Agree; Neutral; Disagree; Strongly disagree | EOT only |
| After I started using the air quality monitor, I | Didn’t change any of my marijuana smoking habits; I thought more about changing my marijuana smoking habits; I smoked less marijuana inside my home; I smoked all of my marijuana outside my home (made my home marijuana smoke free) | EOT only |
| After I started using the air quality monitor, I | Didn’t change any of my tobacco smoking habits; I thought more about changing my tobacco smoking habits; I smoked less tobacco inside my home; I smoked all of my tobacco outside my home (made my home tobacco smoke free) | EOT only; If marijuana and tobacco use |
| After the monitor was installed, other people smoked less marijuana inside my home | Strongly agree; Somewhat agree; Neutral; Somewhat disagree; Strongly disagree | EOT only |
| After the monitor was installed, other people smoked less tobacco inside my home | Strongly agree; Somewhat agree; Neutral; Somewhat disagree; Strongly disagree | EOT only |
| The air monitor caused me to discuss smoking more with the other people living in the home | Strongly agree; Somewhat agree; Neutral; Somewhat disagree; Strongly disagree | EOT only |
| The air monitor caused me to discuss smoking more with the other people visiting the home | Strongly agree; Somewhat agree; Neutral; Somewhat disagree; Strongly disagree | EOT only |
| Using the air monitor was helpful in family conversations about changing indoor smoking rules to protect children from secondhand smoke | Strongly agree; Somewhat agree; Neutral; Somewhat disagree; Strongly disagree | EOT only |
| I would recommend the air monitor to other smokers who are trying to reduce the amount of secondhand smoke in their home | Strongly agree; Somewhat agree; Neutral; Somewhat disagree; Strongly disagree | EOT only |
| I would recommend the air monitor to someone trying to quit or reduce the amount of marijuana they smoke | Strongly agree; Somewhat agree; Neutral; Somewhat disagree; Strongly disagree | EOT only |
| I would recommend the air monitor to someone trying to quit or reduce the amount of tobacco they smoke | Strongly agree; Somewhat agree; Neutral; Somewhat disagree; Strongly disagree | EOT only; If marijuana and tobacco use |
| Which of the following best describes how frequently you reviewed your air monitor measures? | Multiple times per day; About once per day; A few times per week; Once per week; Almost never | EOT only |
| Which of the following best describes how often you looked at your air quality using the monitor or app? | I looked at it more at the beginning of the study; I looked at it more at the end of the study; I looked at it the same amount of time over the entire week study; I didn’t look at it at all | EOT only |
